# Supplementary material for: Learning curve and surgical outcome of robotic assisted colorectal surgery with ERAS program
Source: Sci Rep. 2022 Nov 29;12:20566. doi: 10.1038/s41598-022-24665-w (PMC9709162; doi:10.1038/s41598-022-24665-w)
Supplement: Supplementary file 1 — Supplementary Information 1. [file 41598_2022_24665_MOESM1_ESM.zip › LSC Raw data-Ñ[▒K/054/P(2)_ñw╝╨░O▒Kñσ.pdf]

[redacted]

檢驗(查)報告

申請時間：2019/11/05 09:31 簽收時間：2019/11/05 14:05 檢體 ：Pathology
診斷醫師：侯泰成 傅景佟 報告時間：2019/11/08 09:52 病理號：S0839348

```
Pathologic diagnosis:
Colon, 45 cm from anal verge, polypectomy ---- Hyperplastic polyp.

Gross description:
The specimen consists of a piece of tan soft tissue, 0.2x0.1x0.1 cm.
All for section.

Microscopic description:
Section shows colon mucosa with saw-like proliferation of
superficial mucosal glands without dysplasia.
#T-59300_2 #M-72040_2 0 1340 000000
```

已電子簽章

敬告：本單僅供醫師參考使用，不具任何申請效力！！
請病患妥為保管，若不慎遺失需自負個人資料外洩責任！
